# Case Report: Myelin Oligodendrocyte Glycoprotein Antibody-Associated Disorder Masquerading as Multiple Sclerosis: An Under-Recognized Entity?

**DOI:** 10.3389/fimmu.2021.671425

**Published:** 2021-06-17

**Authors:** Yang Zheng, Meng-Ting Cai, Er-Chuang Li, Wei Fang, Chun-Hong Shen, Yin-Xi Zhang

**Affiliations:** ^1^ Department of Neurology, Zhejiang Provincial Hospital of Chinese Medicine, Hangzhou, China; ^2^ Department of Neurology, Second Affiliated Hospital School of Medicine Zhejiang University, Hangzhou, China; ^3^ Department of Neurology, Fourth Affiliated Hospital School of Medicine Zhejiang University, Yiwu, China

**Keywords:** case report, myelin oligodendrocyte glycoprotein antibody, multiple sclerosis, myelitis lesions, neuroimmunological disease

## Abstract

Myelin oligodendrocyte glycoprotein (MOG) antibody-associated disease (MOGAD) covers a wide spectrum of manifestations and is defined by the presence of MOG seropositivity. However, in a proportion of patients, there may be an overlap in some of the clinical and radiological manifestations between MOGAD and multiple sclerosis (MS). Being wary of this entity is critical to ensure appropriate therapy. Herein, we present a case with recurrent episodes of short-segment myelitis typical for multiple sclerosis, but later diagnosed as MOGAD by MOG antibody seropositivity. This case, along with previous reports, highlights an increasingly recognized subgroup in MOGAD with initial clinical phenotypes suggestive of MS, but later showing a disease course and therapeutic response compatible with MOGAD. Given the potential overlap of some clinical phenotypes in patients with MS and those with MOGAD, we recommend MOG antibody testing in all patients with recurrent short-segment myelitis, conus medullaris involvement, and those who demonstrated steroid dependence.

## Introduction

**Figure 1:**
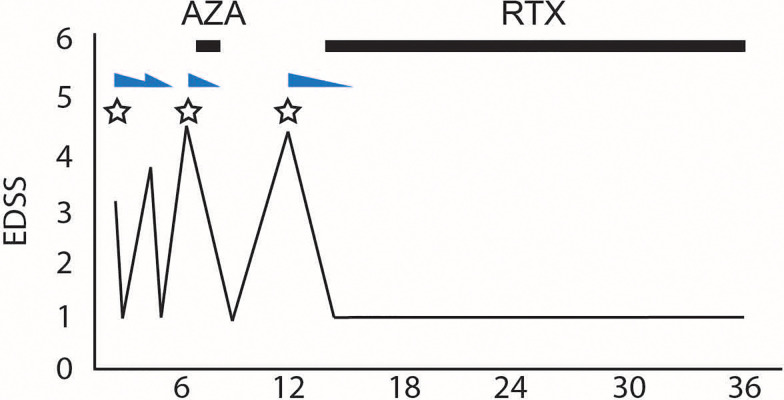
Clinical course and treatment in the case. The x-axis indicates the number of months after disease onset. The y-axis denotes the clinical course documented by EDSS scores. Relapses are indicated by stars. Steroid use and tapering are depicted with blue right-angled triangles. Immunosuppressant use is depicted with blue bars. AZA, azathioprine; EDSS, Expanded Disability Status Scale; RTX, rituximab.

Myelin oligodendrocyte glycoprotein (MOG) antibody-associated disease (MOGAD) is increasingly recognized as a distinct disease entity, yet with a wide spectrum of presentations (1). Herein, we report a case with findings initially concerning for multiple sclerosis (MS) yet subsequently diagnosed with MOGAD (**Figure 1**). Though rare, it is now recognized that patients with MOGAD could have typical MS attacks at onset (2–4). The major challenge, however, lies in the early recognition and correct diagnosis of such patients for an appropriate therapeutic strategy. Our case argued the importance and proposed the rationale for a MOG antibody screen in selected patients with clinical phenotypes suggestive of MS. We also discussed rituximab as a therapeutic option in such patients.

## Case Description

**Figure 2:**
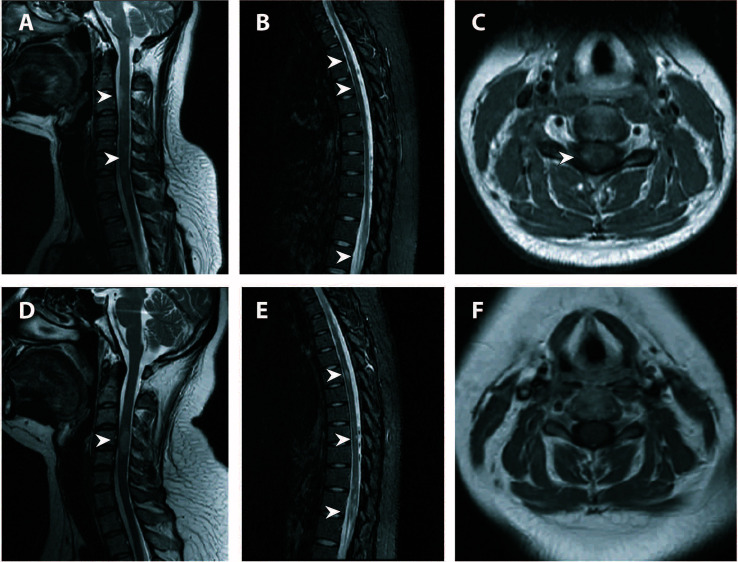
Spinal MRI of the first and third attack. **(A–C)** Cervical (T2-weighted imaging) and thoracic (fat-saturated T2-weighted imaging) spinal MRI of the initial attack revealed multiple T2-hyperintense lesions throughout the spinal cord. Axial fat-saturated T1-weighted imaging with contrast enhancement showed eccentric lesions with patchy enhancement. **(D–F)** Repeat spinal MRI showed new lesions along the cervical and thoracic cord with resolution or attenuation of previous lesions. No enhancement was seen on axial fat-saturated T1-weighted imaging. Lesions were indicated by white arrowheads. MRI, magnetic resonance imaging.

A 25-year-old female presented with a 2-month history of progressive ascending paresthesia with incomplete bladder emptying. On examination, the patient exhibited pain and temperature sensory loss, mild weakness bilaterally (Medical Research Council: 4/5 in right leg; 4+/5 in left leg; 4+/5 in left upper extremity), with an exaggerated deep tendon reflexes and positive Babinski signs bilaterally. Routine workup including complete blood count, basic metabolic panel, and serum autoimmune panel were normal. Spinal magnetic resonance imaging (MRI) with contrast were obtained, revealing multiple T1-isointense and T2-hyperintense patchy lesions along the cervical, thoracic, lumbar segments, each extending over one to two vertebral segments. There was mild swelling of the conus medullaris (**Figures 2A–C**). The lesions were characterized by an unclear demarcation, eccentric axial location, and vague enhancement. MRI of the brain with enhancement and spinal magnetic resonance angiography (MRA) were unremarkable. Cerebrospinal fluid (CSF) analysis demonstrated normal white cell count, glucose, and protein. Infectious tests were negative. Immunoglobulin G (IgG) index was 0.54. Oligoclonal bands (OCB) were positive.

Based on her clinical course, OCB positivity, lack of CSF pleocytosis, and radiological findings, a working diagnosis of inflammatory/demyelinating myelitis was favored. The patient received empirical steroid pulse therapy (500 mg/d intravenous) for 5 days with a gradual oral tapering, upon which the weakness significantly ameliorated and the paraesthesia improved. However, the patient returned to the clinic when on 10 mg/d prednisolone and reported aggravation of numbness and weakness. Repeat thoracic MRI showed multiple patchy short-segment lesions and no new lesions were detected. The dose of prednisolone was increased to 25 mg/d, upon which, the symptoms alleviated.

**Figure 3:**
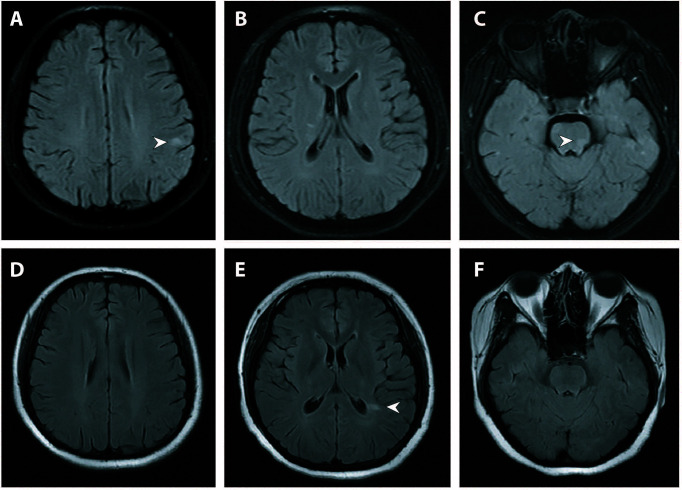
Brain MRI of the second and third attack. **(A–C)** Brain MRI (fat-saturated T2-weighted fluid attenuated inversion recovery imaging) at the second attack showed ovoid juxtacortical and pontine lesions. **(D–F)** Repeat MRI (T2-weighted fluid attenuated inversion recovery imaging) at the third attack after steroid cessation showed new lesions adjacent to the left posterior horn of the lateral ventricle and resolution of juxtacortical and pontine lesions. Lesions were indicated by white arrowheads. MRI, magnetic resonance imaging.

Unfortunately, 2 months after steroid cessation, the patient had another attack with leg numbness consistent with myelitis. Repeat spinal MRI revealed new patchy lesions and resolution of previous lesions. Brain MRI this time showed multiple juxtacortical and brainstem lesions with an ovoid shape (**Figures 3A–C**), which was not present at her initial presentation. Till now, the patient reached both the temporal and spatial dissemination criteria of clinically definite MS. Another course of intravenous methylprednisolone (500 mg/d) was initiated with a complete remission of symptoms. Given the lack of officially approved disease modifying therapy (DMT) drugs in China at that time, azathioprine (AZA) with a dose of 50 mg twice per day was initiated for disease prevention.

Six months later after the second attack, upon cessation of AZA treatment herself, the patient returned with the third relapse. The patient was readmitted for further evaluation. Serum MOG antibody testing (commercially available fixed cell-based assay employing full-length MOG antigen [Euroimmun]) returned positive with a titer of 1:32. Serum aquaporin 4 antibody testing was negative. Repeat spinal MRI revealed co-existence of both new lesions and old lesions along the cervical and thoracic cords (**Figures 2D–F**). Repeat brain MRI showed new lesions adjacent to the posterior horn of the lateral ventricle and resolution of the subcortical and brainstem lesions (**Figures 3D–F**).

### Diagnostic Assessment

Given the constellation of recurrent short-segment myelitis, serum MOG antibody positivity and prominent therapeutic response to and dependence on steroid, the diagnosis of MOGAD was made. We initiated methylprednisolone pulse therapy (1000 mg for 5 days) followed by a slow tapering over 6 months. The maintenance regimen was discussed with the patient. Lower-dose rituximab (RTX) was chosen considering its relative efficacy and safety profile (5, 6). RTX was started with an initial dose of 1000 mg and a maintenance dose of 500 mg every half year. During the 2-year follow-up, the patient remained relapse-free, and no adverse events were observed. The patient declined repeat brain or spinal imaging due to an absence of clinical signs or symptoms.

## Discussion

Our case, along with an increasing number of reports (**Table 1**), suggests a proportion of MOGAD with initial phenotypes suggestive of MS (2–4). The presence of lesions with shapes and distributions compatible with MS, as well as her OCB positivity led to the initial misdiagnosis of MS. However, her serum MOG antibody seropositivity (cell-based assay), the suitable clinical picture of recurrent myelitis involving the conus medullaris, and a substantial dependency on steroids all supported the final diagnosis of MOGAD.

**Table 1:** Cases with MOG-AD mimicking typical MS.

No	Sex	Onset, y	Initial syndrome	Spinal MRI	Cerebral MRI lesions	Intrathecal OCBs	MS criteria (last F/U)	Other autoimmune conditions	Past DMTs with unsatisfied response	Drugs with a favorable response
1. Spadaro et al. (2)	F	9	Brainstem	Short-segment myelitis	Periventricular, cortical/juxtacortical, and pontomedullary	Yes	Yes	Uveitis	IFN, GLAT, IVIg	PLEX
2. Spadaro et al. (2)	F	18	Myelitis	LETM, conus medullaris involvement	Periventricular, cortical/juxtacortical, pontomedullary lesions and cerebellar	Yes	Yes	None	GLAT, FTY, NAT	RTX
3. Spadaro et al. (2)	F	47	Myelitis	LETM	Periventricular, cortical/juxtacortical, pontomedullary esions, and cerebellar	Yes	Yes	Hashimoto, uveitis	GLAT, FTY, IFN, DMF	NAT, PLEX
4. Spadaro et al. (2)	M	37	Myelitis	Short-segment myelitis	Periventricular, cortical/juxtacortical, and cerebellar	Yes	Yes	Graves disease	IFN, GLAT	None
5. Spadaro et al. (2)	M	22	Myelitis	Short-segment myelitis	Periventricular, cortical/juxtacortical, and pontomedullary	Yes	Yes	None	IFN	NAT, RTX, PLEX
6. Breza et al. (3)	M	31	Myelitis	Short-segment myelitis, conus medullaris involvement	Periventricular	Yes	Yes	None	None	None
7. Dolbec et al. (4)	F	26	Myelitis	Short-segment myelitis	Periventricular, and cortical/juxtacortical	Yes	Yes	None	IFN	RTX
8 (index)	F	25	Myelitis	Short-segment myelitis, conus medullaris involvement	Periventricular, cortical/juxtacortical, and pontomedullary	Yes	Yes	No	AZA	RTX

AZA, azathioprine; DMT, disease modifying therapy; F, female; F/U, follow-up; FTY, fingolimod; GLAT, glatiramer acetate; IFN, interferon; IVIg, intravenous immunoglobulin; LETM, longitudinal extensive transverse myelitis; M, male; MOG-AD, myelin oligodendrocyte glycoprotein antibody-associated disease; MRI, magnetic resonance imaging; MS, multiple sclerosis; NAT, natalizumab; OCB, oligoclonal band; PLEX, plasma exchange; RTX, rituximab.

Though considered as distinct disorders, manifestations of MS and MOGAD may overlap. Among them, short-segment myelitis constitutes the initial presentation in more than half of multiple sclerosis (MS), up to 53% of MOGAD (1, 7–9) and 15% of neuromyelitis optica spectrum disorder (10, 11). In addition, around 6% to 17% of MOGAD patients have positive CSF OCB (12, 13) and 33% may fulfill the McDonald diagnostic criteria (1, 9). Also, given their overlapping presentations, differentiation between the two disorders is extremely important based mainly on two reasons: 1) maintenance therapy of MS and MOGAD differs greatly: on the one hand, DMT are mainly used in patients with MS; on the other, patients with MOGAD are particularly responsive to antibody-depleting treatments, but may even deteriorate with DMT (1, 14); 2) MOGAD is associated with a high risk of flare-ups after cessation of steroid treatment and may thus require close monitoring and careful steroid tapering at least over 6 months (14).

The conundrum, however, lies in the selection of patients based on the trade-off between the highest diagnostic yield and unnecessary testing. The presence of short-segment myelitis or intrathecally restricted OCBs should not exclude a diagnosis of MOGAD. Further, serum MOG antibody testing is warranted in patients with the following features, even if the McDonald criteria were fulfilled: 1) recurrent myelitis with or without brain lesions; 2) conus medullaris involvement, especially if present at onset; 3) unsatisfied disease control with steroid or DMT, i.e., frequent flare-ups during steroid tapering or after steroid cessation, or relapses even during DMT use (7, 9, 10). Notably, the clinical clues become more important with a low-titer MOG antibody testing result. In our case, a titer of 1:32 with fixed assays needs to be interpreted with caution (15). It is critical to evaluate the clinical phenotype and diagnosis based solely on antibody testing should be avoided (16). It is the constellation of recurrent myelitis, lack of brain lesions at the first episode, involvement of conus medullaris, and a remarkable steroid dependence that led to the final diagnosis of MOGAD.

The emerging recognition of MOG patients with MS-like phenotypes also holds therapeutic implications. MOGAD exhibits a poor response to DMTs, including interferon-beta and glatiramer, further supporting the notion that MOGAD and MS belong to distinct entities. Rather, a heterogeneous efficacy could be achieved with mycophenolate mofetil, AZA, RTX, or oral prednisolone (17). In particular, RTX, a B-cell depleting agent, was reported to reduce relapse rate by around 40% when used in treatment naive patients (18). However, around 80% of relapses continued despite robust B-cell depletion. Therefore, identifying those RTX-responsive patients when determining the therapeutic strategy would be of great value. Interestingly, in the eight patients with clinical phenotypes suggestive of MS but ultimately diagnosed with MOGAD (**Table 1**), there is a consistent response in all four patients treated with RTX. It may offer preliminary indications that MOGAD patients with MS-like presentations might be optimal candidates for RTX treatments.

Despite the efficacy of RTX in a proportion of MOGAD patients, there lacks a consensus on its standard use. Notably, in our case, we chose a lower-dose regimen of RTX (two initial infusions of 500 mg given a fortnight apart and subsequent infusions of 500 mg every 6 months). The regimen of 500 to 1,000 mg is originally adopted from clinical trials in rheumatoid arthritis (19, 20). One reason to choose the lower end of the spectrum lies in the relative smaller body habitus of the Asian population, indicating a smaller body surface area and a lower tolerability to RTX (21). Furthermore, previous literature also noted no significant difference in clinical response with high-dose (2 × 1,000 mg) and lower-dose (2 × 500 mg) RTX regimens in rheumatoid arthritis (19). In neuroinflammatory diseases, such as neuromyelitis optica, responsiveness could be also achieved with an even lower dose of RTX (100mg weekly for 4 weeks) in Chinese patients (21). Our patient with MOGAD further supports the notion that therapeutic objectives might be achieved with a lower-dose RTX regimen in Chinese patients.

In conclusion, our case highlights the importance in differentiating between MS and MOGAD in patients with recurrent short-segment myelitis, especially with conus medullaris involvement and unsatisfied therapeutic response. Serum MOG antibody testing would aid in the correct diagnosis and initiation of appropriate treatment.

## Data Availability Statement

The original contributions presented in the study are included in the article/supplementary material. Further inquiries can be directed to the corresponding author.

## Ethics Statement

Written informed consent was obtained from the individual for the publication of any potentially identifiable images or data included in this article.

## Author Contributions

YZ and M-TC contributed to design and concept of the study, and drafting the initial manuscript. E-CL, WF and C-HS contributed to acquisition and analysis of the clinical data. Y-XZ contributed to revising the manuscript for intellectual content. All authors contributed to the article and approved the submitted version.

## Conflict of Interest

The authors declare that the research was conducted in the absence of any commercial or financial relationships that could be construed as a potential conflict of interest.
